# Cecum cancer underlying appendicular abscess. Case report and review of literature

**DOI:** 10.1186/1749-7922-1-11

**Published:** 2006-04-04

**Authors:** Irene Fiume, Vincenzo Napolitano, Gianmattia Del Genio, Alfredo Allaria, Alberto Del Genio

**Affiliations:** 1 First Division of General and Gastrointestinal Surgery, Second University of Naples, Italy

## Introduction

Peri-appendicular abscess is usually due to primary appendicitis or it may be caused by various pathological conditions: inflammatory, infective, neoplastic, immunological, ischemic, occlusive (table 1).

**Table 1:** Causes of appendicitis

**PRIMARY CAUSES OF APPENDICITIS**
**Idiopathic lymphatic hyperplasia ** **Fecalith obstruction**
**SECONDARY CAUSES OF APPENDICITIS**
**Inflammatory**
Crohn's disease
Ulcerative Colitis
Sarcoidosis
Granulomatous appendicitis
**Infective**
Bacterial (Tuberculosis, Typhus, Actinomycosis, *E. coli *and *B. fragilis*, *Pseudomonas*, *Yersinia*, *Eikenella corrodens *infections)
Viral (Adenovirus, Cytomegalovirus infections)
Fungal (Aspergillosis, Histoplasmosis)
Parasitic (*Enterobius vermicularis*, *Strongyloides stercoralis*, *Entamoeba histolytica *infections, Ascariasis, Schistosomiasis, Cryptosporidiosis, Taeniasis)
**Neoplastic**
Primary neoplasm (Adenoma, Mucinous cystadenomas, Cystadeno-carcinomas, Carcinoid tumors, Mesenchymal tumors)
Metastasis from other organs (stomach, lung, femal genital tract)
Lymphoma-leukemia
Endometriosis
**Ischemic**
Vascular occlusion for thrombosis, vasculitis
**Neurogenic**
Neural proliferations and/or increased number of endocrine cells
**Foreign body obstruction**
Small chicken and fish bones, portion of wire, nail, cherry stone, piece of glass, dislocated intrauterine contraceptive device, dislocated biliary stent

We present a case of right iliac fossa abscess, initially diagnosed as a complication of acute appendicitis that, at surgical exploration, was proven to be produced by a cecal tumour causing appendicitis for an obstructive mechanism.

## Case report

A 63-year-old male patient in reasonably good health suddenly suffered from fever (>38°C) and a painful right iliac fossa tumefaction; no other gastrointestinal problems were noted. On clinical examination, a mass of about 5 cm was palpable in correspondence of right iliac fossa and appeared hard, not reducible and fixed to the parietal muscle.

Laboratory data showed mild anaemia (haemoglobin: 10.7 g/dl, hematocrit: 31%) and elevated erythrocyte sedimentation rate (74 mm/hr) as pathological findings. Alvarado score was 6 and therefore a parenteral antimicrobial therapy was administered.

**Figure 1:**
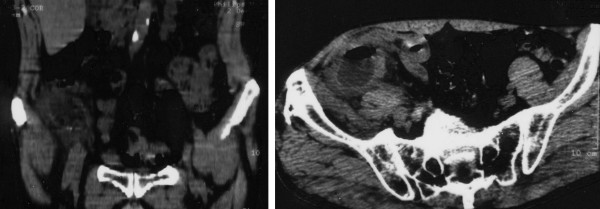
Abdomino-pelvic CT scan reveals a 6 × 8 cm right iliac fossa abscess with a remarkable wall thickening of the adjacent cecum.

Abdominal ultrasound examination evidenced an abscess in the right lower quadrant, with heterogenic echotexture and a thickening of the ileocecal tract. Abdominal computerized tomography (CT), confirmed the presence of a complex, predominantly cystic, mass of large size (6 × 8 cm) with heterogeneous, mainly peripheral enhancement (figure 1). The adjacent cecum had its wall thickened and it was not possible to differentiate the appendix separately from the mass. Homolateral inguinal reactive lymphadenopathy was also present. The patient failed to respond to the initial conservative management, which consisted of intravenous fluids and triple antibiotic therapy with cefotaxime, gentamicin and metronidazole, without any improvement of pain and fever. At a further ultrasound examination, the mass appeared not modified.

Because of worsening abdominal pain and clinical signs of right iliac fossa peritonitis, the patient was not submitted to contrast enema and colonoscopy to avoid the risk of extensive peritoneal contamination for abscess rupture or exacerbation of bowel perforation with severe diffuse peritonitis and was directly operated on.

## Surgical procedure

Exploratory laparotomy revealed a voluminous cecal mass fixed to the abdominal wall, covering a purulent, loculated abscess cavity that was copiously irrigated and drained. A thick and dilated appendix was finally evidenced strictly adherent to the cecal mass that appeared quite suspicious. Careful inspection of liver, spleen, peritoneal cavity and mesentery was done. The lesion was considered resectable and a right-sided hemicolectomy was performed.

## Results

Gross examination of the resected specimen showed an ileocecal valve tumour with ulcerated aspect extending to the fundus of cecum. The histopathology revealed a well-differentiated adenocarcinoma invading the muscularis propria. No metastatic lymph nodes were found (B2 stage in the Astler-Coller classification). Resection margins were free. Marked peri-appendiceal inflammation significant for appendiceal perforation was evidenced. Appendicitis and perforation originated from chronic obstruction of the base of appendix caused by the cecal neoplasm.

The patient underwent adjuvant chemotherapy with fluorouracil and levo-folinic acid. At a 6 month follow-up no sign of recurrence was evidenced.

## Discussion

Pathophysiologically, the relations between cecum cancer and appendicular abscess may be the followings: 1) cecum cancer and appendicular abscess may be two co-existing and independent affections, 2) cecal neoplastic lesion may cause appendicitis by mechanical obstruction at the orifice of the vermiform appendix, 3) adenocarcinoma of cecum may present clinically as appendiceal abscess due to transmural invasion with perforation.

Colonic perforation due to carcinoma may occur at the tumour, either proximal or distal to the neoplastic lesion [1]. The perforation may be due to transmural invasion of the neoplasia or to obstructive mechanism. In the case we describe, the perforation occurred distal to the cancer, in correspondence of the appendix. The spreading ileocecal valve neoplasia reached the orifice of the appendix and caused the appendiceal occlusion and perforation.

Patients with perforated cancer are at risk of diffusion of cancer cells within the abdomen and pelvis and consequently of peritoneal carcinomatosis [2]. However, different clinical reports show that the presence of perforation doesn't necessarily predispose a poor prognosis and long-term survivals depend on tumour stages without significant difference between perforated and uncomplicated cancers [3-5]. Although the site of perforation doesn't appear to impact the 5-year survival, a few Authors describe a worse long-term outcome related to perforation proximal to a cancer [1,6]. 

Sonography and computed tomography show a high degree of accuracy in detecting the presence of an abscess [7]. The radiologic appearance of cecal wall thickening associated with an abscess may indicate an involvement of cecum that may be primary (diverticulitis, inflammatory diseases, neoplasia, infections) or secondary to appendicitis.

Preoperative colonoscopy in presence of cecal abscess and inflammation may be hazardous and be at risk of complications such as diffuse peritonitis for abscess rupture or exacerbation of bowel perforation, development of liver abscess [8-10]. Also barium enema, commonly used in the past, it is no longer recommended because of the risk of extravasation of contrast material if perforation has occurred [9]. 

The treatment of appendiceal abscesses is still discussed and many different approaches are nowadays adopted. Expectant management, consisting of intravenous antibiotics, percutaneous drainage and interval appendectomy at a later date, is gaining a wide consent as it seems associated with less morbidity and shorter overall hospital stay [11,12]. However, malignancy is a substantial risk in elderly patients. Carcinoma masquerading as appendicitis occurs more often than is generally realized and will be seen more frequently as aging population increases [13]. The percentage of patients presenting with appendicitis that also have colonic carcinomas is ranging from 1, 8 to 8% according various Authors [14-16].

Therefore, an aggressive surgical approach of cecal masses, with intra-operative abscess drainage and resection should be performed in selected cases to avoid the risk of delaying the diagnosis and unrecognising malignant lesion as well as to achieve a safe and adequate treatment [17]. Careful intraoperative assessment, including evaluation of the tract of the bowel involved by the inflammation, inspection and palpation of the liver, resection and examination of the specimen is essential to identify malignancy [16]. In the suspect of neoplastic lesion of cecum or appendix, right hemicolectomy is the procedure of choice to make a correct diagnosis and to avoid the risk of leaving residual tissue [16,18].
